# Diabetes Mortality and Morbidity Trends and Related Risk Factors in Iranian Adults: An Appraisal via Current Data

**Published:** 2018-10

**Authors:** Yousef Veisani, Salman Khazaei, Ensiyeh Jenabi, Ali Delpisheh

**Affiliations:** 1 Psychosocial Injuries Research Center, Ilam University of Medical Sciences, Ilam, Iran. 69311-63545. Tel: +98 918170082. Fax: +98 8432227132. E-mail: yousefveisani@yahoo.com.; 2 Department of Epidemiology, School of Public Health, Hamadan University of Medical Sciences, Hamadan, Iran. 65158-838695. Tel: +98 8138380548. Fax: +98 8138380130. E-mail: salman.khazaei61@gmail.com.; 3 Pediatric Developmental Disorders Research Center, Hamadan University of Medical Sciences, Hamadan, Iran. 65158-838695. Tel: +98 8138380548. Fax: +98 8138380130. E-mail: en.jenabi@yahoo.com.; 4 Department of Clinical Epidemiology, Ilam University of Medical Sciences, Ilam, Iran. 69311-63545. Tel: +98 9121307577. Fax: +98 8432227132. E-mail: alidelpisheh@yahoo.com.

Diabetes is among the 4 noncommunicable diseases (NCDs) assigned for eradication by the world leaders in the 2011 Political Declaration on the Prevention and Control of NCDs.^1^^, ^^2^ Concern regarding the prevalence rate of diabetes and its resultant mortalities has increased in the last 2 decades.^3^ It has been documented that regular physical activity and weight control can reduce the risk of diabetes.^4^

In this study, we assessed the current data on diabetes and its related risk factors in Iran based on the diabetes country profiles 2016 of the World Health Organization (WHO).^5^ Overall, 9309 people died from diabetes in Iran in 2015, which accounts for only 2% of all NCDs. Additionally, the age-standardized mortality rate of diabetes had a rise in 2015 compared with previous years^6^ (Table 1). 

Indeed, the burden of diabetes is extremely high because it is associated with premature death from cardiovascular disease, cancer, and non-cardiovascular non-cancer causes. Furthermore, diabetes can be considered the original cause and promoter of ischemic heart disease.

**Figure 1:**
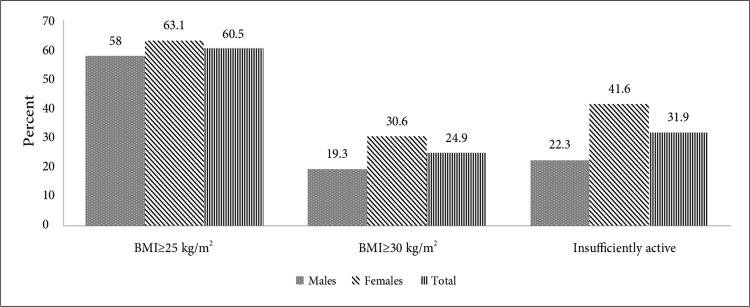
Common risk factors for diabetes among Iranian adults (2016)^5^

The frequency of the important risk factors of diabetes in Iran is depicted in Figure 1.^5^ In 2016, the prevalence rates of physical inactivity, overweight, and obesity were reported to be 31.9%, 60.5%, and 24.9%, respectively. In all the related risk factors, a higher prevalence rate was reported in females.

**Figure 2:**
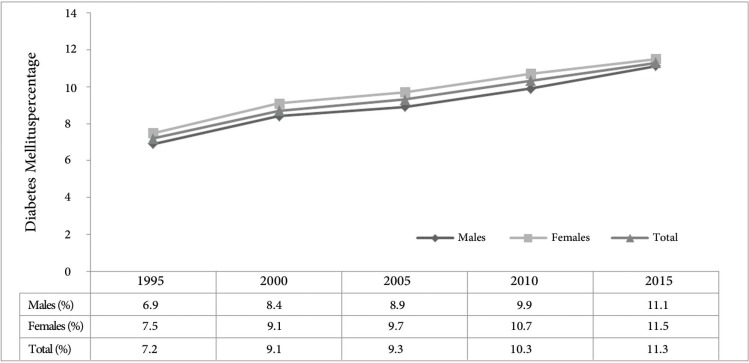
Trends in the age-standardized prevalence of diabetes by gender and in total in total population of Iran (1995–2015)^5^

As is shown in Figure 2, the prevalence rate of diabetes (age-standardized) exhibits a steady rise by about 2.6% in this 15-year period, growing from 8.7% in 2000 to 11.3% in 2015.^5^

**Table 1:** Trends in the age-standardized prevalence of NCDs and diabetes in Iran (2000–2015)^6^

Year	Total Population	NCDs		Diabetes Mellitus	
		Age-Standardized Mortality Rate (per 100,000 population)	Total NCD Deaths	Age-Standardized Mortality Rate by Cause (per 100,000 population)	Number of Diabetes- Related Deaths
2015	79 109 000	570.0	297 900	11.3	9309
2010	75 149 000	605.4	289 400	10.3	9033
2000	65 392 000	737.3	242 000	8.7	8807

NCDS, Noncommunicable diseases

BMI, Body mass index

In conclusion, our results show that the age-standardized mortality rate of diabetes in Iran has been steadily rising, totally and in both genders, in the last 2 decades. Moreover, an epidemic picture can be observed in the risk factors related to diabetes. The interesting result from this study with respect to the 3 salient risk factors related to diabetes (i.e., insufficient physical activity, overweight, and obesity) is that 42.9% of the females and 24.1% of the males were classified as insufficiently physically active. This finding is concordant with previous studies showing that insufficient physical activity, overweight, and obesity are significantly linked to diabetes.^7^^, ^^8^ Therefore, diabetes together with its related factors is a great concern for Iranian adults in the coming years. To overcome this serious threat, policymakers should focus on operational policy/strategy/action plans aimed at reducing overweight and obesity in the Iranian adult population. 
